# Correlation between 3D scanner image and MRI for tracking volume changes in head and neck cancer patients

**DOI:** 10.1002/acm2.13181

**Published:** 2021-02-01

**Authors:** Jung‐in Kim, Joo‐Hyun Chung, Ohyun Kwon, Jong Min Park, Hong‐Gyun Wu

**Affiliations:** ^1^ Department of Radiation Oncology Seoul National University Hospital Seoul Korea; ^2^ Biomedical Research Institute Seoul National University Hospital Seoul Korea; ^3^ Institute of Radiation Medicine Seoul National University Medical Research Center Seoul Korea; ^4^ Robotics Research Laboratory for Extreme Environments Advanced Institutes of Convergence Technology Suwon Korea; ^5^ Department of Radiation Oncology Seoul National University College of Medicine Seoul Korea; ^6^ Cancer Research Institute Seoul National University College of Medicine Seoul Republic of Korea

**Keywords:** 3D scanner, adaptive radiotherapy, head and neck cancer, surface imaging, volume changes

## Abstract

**Introduction:**

We investigated the correlation between optical surface imaging using a three‐dimensional (3D) scanner and magnetic resonance imaging (MRI) for suggesting feasibility in the clinical process of tracking volume changes in head and neck patients during radiation treatment.

**Methods:**

Ten patients were divided into two groups depending on the location of their tumor (i.e., right or left side). With weekly imaging data, the change in volume based on MRI was evaluated during the treatment course. Four volumes of interest (VOIs) were calculated on the 3D surface image of the facial and cervical areas using an optical 3D scanner, and the correlation between volumetric parameters were analyzed.

**Results:**

The target volume changed significantly overall for both groups. The changes parotid volume reduced by up to 3.8% and 28.0% for groups A (right side) and B (left side), respectively. In Group A, VOI 1 on the facial area and VOI 3 on the cervical area decreased gradually during the treatment course by up to 3.3% and 10.7%, respectively. In Group B, only VOI 4 decreased gradually during the treatment course and reduced by up to 9.2%. In group A, the change in target volume correlated strongly with right‐side parotid, VOI 1, and VOI 3, respectively. The parotid also showed strong correlations with VOIs (*P *< 0.01). The weight loss was strongly correlated with either PTV or parotid without statistical significance (*P *> 0.05). In group B (left side), the change in target volume correlated strongly with each volumetric parameter, including weight loss. For individual patient, PTV showed more correlation with VOIs on the cervical area than VOIs on the facial area.

**Conclusions:**

An optical 3D scanner can be applied to track changes in volume without radiation exposure during treatment and the optical surface image correlated with MRI.

## 1 INTRODUCTION

Head and neck (H&N) cancers are often treated using radiation therapy.^1^ Advanced radiation techniques such as intensity‐modulated radiation therapy (IMRT) and volumetric modulated arc therapy (VMAT) can deliver highly conformal doses to the target volumes while sparing the adjacent normal tissue.^2^ During the course of treatment, patients exhibit significant anatomical changes related to tumors response and weight loss.^3^, ^4^, ^5^ Anatomical changes in both tumor and normal tissues can result in underdosage or dose inhomogeneity for the target and overdosage for the organs at risk (OARs). Wang et al. reported that the parotid and submandibular glands (SMG) shrunk during radiation therapy.^6^ Shreerang et al. investigated the weekly volume changes in target volumes and OARs using repeated computed tomography (CT) scans.^7^ The most significant volumetric changes and dosimetric alterations in tumor volumes and OARs during a course of chemotherapy with IMRT occurred by week 2 of radiotherapy.

Adaptive radiotherapy (ART) is a novel approach in which the treatment plan is adjusted during the course of treatment to account for anatomical changes and improve therapeutic gain. Recently, clinical outcomes have been reported for H&N ART.^8^, ^9^, ^10^ Schwartz et al. performed a trial investigating ART for oropharyngeal squamous cell carcinoma in order to examine toxicity and survival outcomes.^11^, ^12^ They concluded that ART can provide a dosimetric benefit with only one or two re‐planning interruptions and that properly timed re‐planning delivers achievable dosimetric improvement a majority of the time. ART is directly associated with image‐guided radiotherapy (IGRT) using in‐room megavoltage (MV) CT, CT‐on‐rails, or cone‐beam CTs (CBCT) obtained prior to daily treatment.^13^ Currently, adaptive magnetic resonance image‐guided radiation therapy (MR‐IGRT) has been reported and clinically implemented.^14^, ^15^ Acharay et al. reported the first successful clinical application of online adaptive MR‐IGRT.^15^ They concluded that the trigger for re‐optimization should be considered with changes in anatomy because not all patients derive the same benefit from ART. However, there are several limitations to the high cost of installation, the operational experience required by the operator, and in the case of IGRT, additional radiation exposure.

Optical 3D surface scanning systems have been applied to radiation therapy because of their advantages of low cost, accuracy, speed, and flexible handling without unnecessary radiation exposure. All studies on 3D scanning systems only analyzed the accuracy of the patient positioning.^16^, ^17^, ^18^, ^19^ There was no study to volumetric analysis using an optical 3D scanner. In this study, we investigated the feasibility of using optical 3D scanner surface imaging for predicting volume changes during the treatment course and evaluated the correlation between MRI and these images.

## 2 MATERIALS AND METHODS

### 2.A Patient selection and MRI imaging

In this study, ten prospective patients among the H&N cancer patients in our institution were randomly enrolled. It was approved by the institutional review board of Seoul National University College of Medicine/Seoul National University Hospital (IRB No.1505‐055‐671) for this study. All patients in this manuscript have signed informed consent forms and agreed to publish these case details. Patients were divided into two groups depending on the location of their tumor (i.e., right or left side). Table 1 summarizes the patient characteristics. Each patient underwent CT scans with the Brilliance CT Big Bore^TM^ (Phillips, Cleveland, OH, USA) at a slice thickness of 3 mm and treated with a VMAT plan generated with the Eclipse^TM^ system (Varian Medical Systems, Palo Alto, CA, USA). Prescription doses of 67.5 Gy were delivered to the PTV in 30 fractions using 6 MV photon beams of Trilogy^TM^ with the Millennium 120^TM^ MLC (Varian Medical Systems, Palo Alto, CA, USA). The PTV was defined by adding 3‐mm margins in every direction from the clinical target volume (CTV). Ninety‐five percent of the PTV was covered by prescription dose and the OARs did not exceed their respective tolerance doses. The normal tissue tolerance levels followed the recommendation by the Radiation Therapy Oncology Group 0615 protocol (RTOG 0615).^20^ ViewRay™ (ViewRay Inc., Cleveland, OH) has been clinically implemented at our institution. This MR‐IGRT system was used to acquire weekly MR images for volume changes during the treatment course (7 weeks), including follow‐up after treatment. MR images were acquired with true fast imaging with steady state precession (TRUFI) sequence, which is a type of fast gradient echo sequence. The MRI resolution was 1.5 × 1.5 × 1.5 mm^3^ with a typical imaging time of 3 min and a field of view of 50 cm. The MR imaging unit of the system provides a 50‐cm diameter spherical field of view. The target volume and OAR structures were delineated on the weekly MRI by one physician to avoid the interpersonal variation.

**TABLE 1:** Patient characteristics.

Patient group	Sex	Age	Type	Stage	Chemotherapy
A (Right side)	M	63	Tonsil	T2N2b	Yes
	M	77	SMG	T4N1	Yes
	M	61	Tongue	T4aN2c	Yes
	M	57	Tonsil	TcN2c	Yes
	F	50	Parotid_Rt	T4aN0	No
B (Left side)	M	47	Tonsil	T2N2b	Yes
	M	53	Tongue	T1N2b	Yes
	F	43	NPx	T3N2	Yes
	F	41	NPx	T3N2	Yes
	M	76	Parotid_Lt	T4bN2	No

F, female; M, male; SMG, submandibular gland; NPx, Nasopharynx.

### 2.B Surface imaging with the 3D optical scanner

**FIG. 1:**
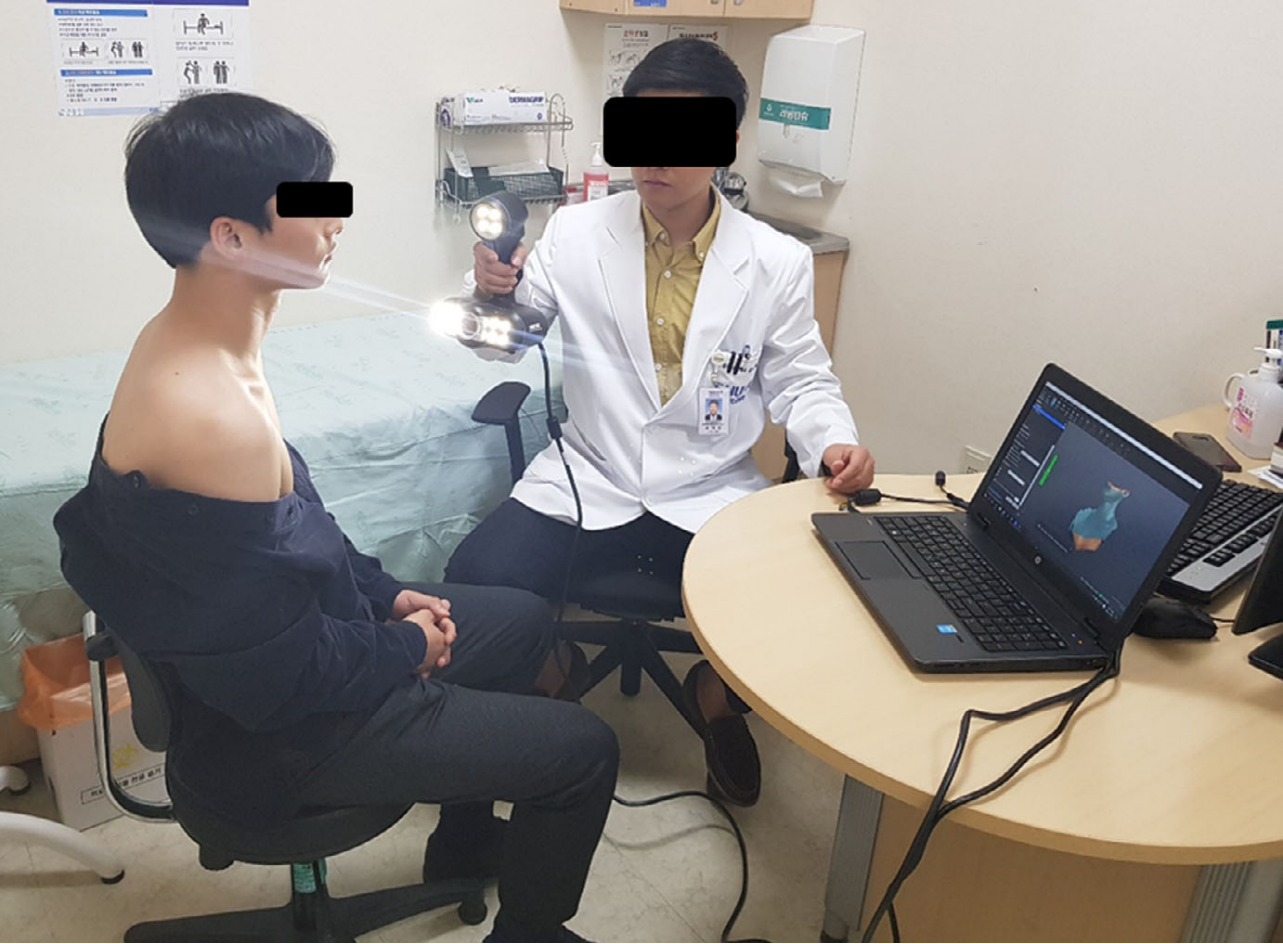
Patient scanning using an optical three‐dimensional scanner.

We used a portable structured‐light 3D scanner, a price of about $ 20 000, weighing 950 g (Go!SCAN 50^TM^, CREAFORM Inc., Canada) to generate high‐resolution 3D images of the subjects (0.5 mm) at the stand‐off distance of 400 mm. The scanner, which has a capable of 550 000 measurements per second, comprised two digital cameras and one digital coler camera, with each camera surrounded by a set of four white light‐emitting diodes (LEDs) and a white light pattern projector. The projector emitted a white light pattern on the object. The pattern distortion on the object was recorded using the three digital cameras. The entire light pattern was used for acquisition. The collected geometry information was used to build the surface with real‐time positioning. The volumetric accuracy was 0.3 mm/m based on the International Organization for Standardization (ISO) 10360 standard.^21^ The scanning images from the well‐calibrated 3D scanner were processed in real‐time using a 3D software platform (VXelements^TM^, CREAFORM Inc., Canada). To ensure reproducibility, the upper half of the body not in a treatment position for each patient was scanned with the same posture weekly; the process took <5 min without analysis. Figure 1 is a picture of the patient scanning using the 3D optical scanner.

### 2.C Evaluation of volume changes

**FIG. 2:**
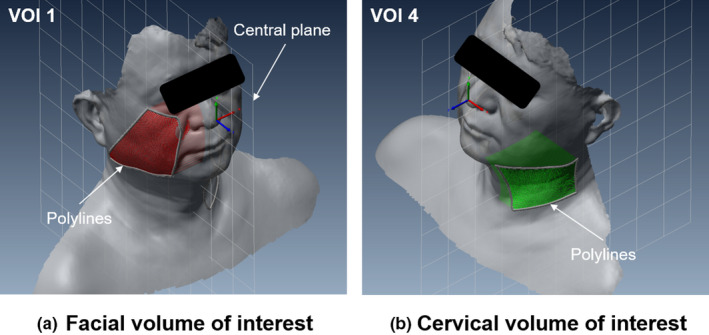
Representative VOIs on a reconstructed surface image for (a) right facial area (VOI 1) and for left cervical areas (VOI 4).

**FIG. 3:**
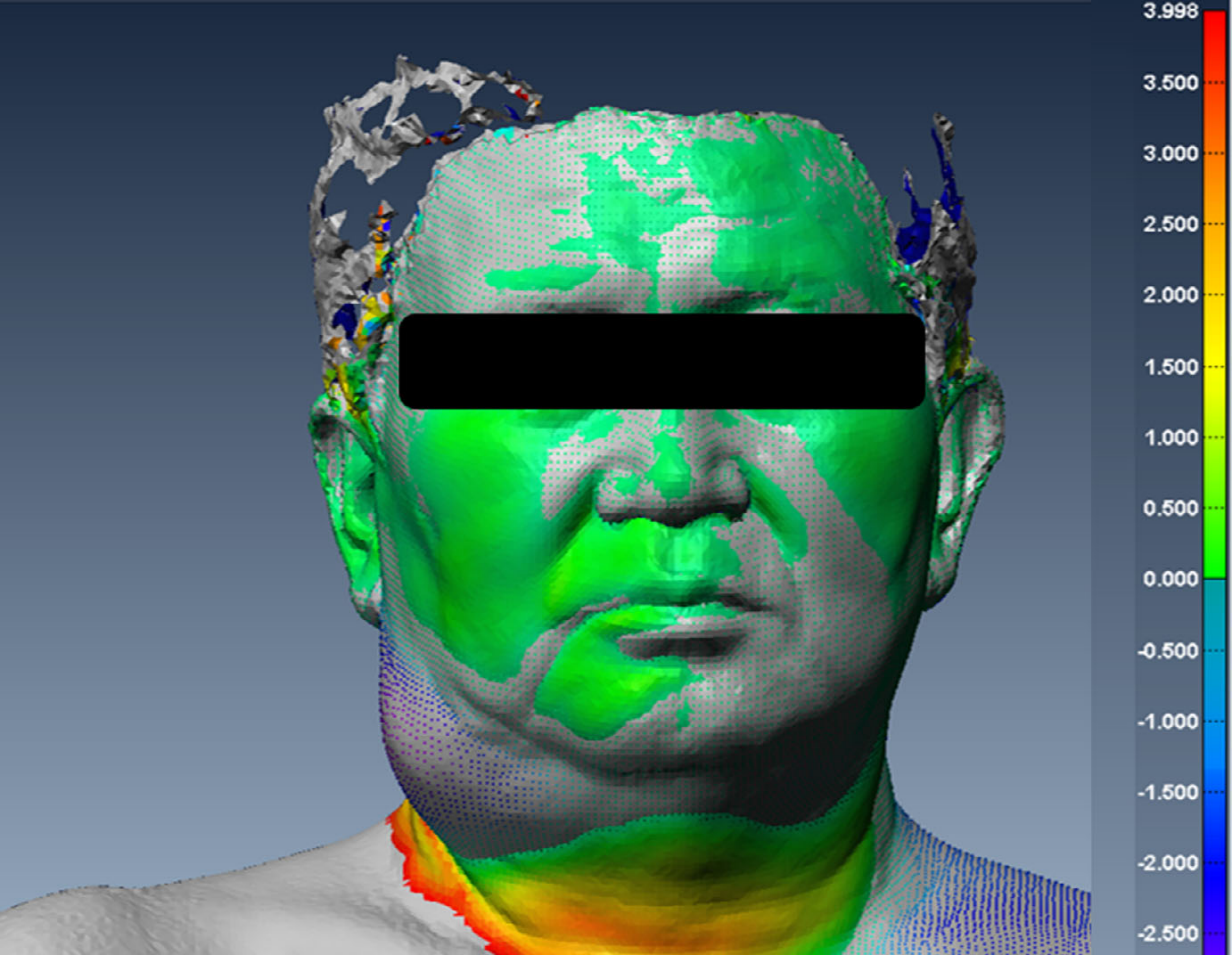
Sample after image registration between three‐dimensional (3D) surface images and the polylines defined on the initial 3D surface image when applied to other 3D surface images for one patient. The color map indicates the difference between the registered 3D surface images.

PTV and parotid volumes were calculated with the MRI to evaluate the changes during the treatment course. The PTV volume was defined from the contoured CTV in the weekly MRI. The volume changes of the spinal cord and brainstem were not included in the analysis. The surface images from the 3D optical scanner were used to envelop the volume and surface area through postprocessing software. The 3D reconstructed polygonal mesh data from VXelements^TM^ were transferred to volume graphic analysis software (PolyWorks Inspector^TM^, InnovMetric Software Inc., Canada). Four volumes of interest (VOIs) on the reconstructed polygonal surface image were defined on the facial and cervical areas. VOI 1 and VOI 2 were divided into right and left sides in the facial area. VOI 3 and VOI 4 were also divided into right and left sides in the cervical area. First, we defined the coordinate system so that the tip of nose was the origin. Then, the central plane was determined along the central axis and we defined the polylines on the surface image of the facial and cervical areas. The polylines for each VOI consisted of several points that could serve as landmarks on the head and neck. Finally, each VOI were defined as the volume between the surface formed by polyline and the central plane as shown in Fig. 2. Each VOI on the reconstructed surface image were calculated to evaluate the changes during the treatment course. The polylines defined in the initial 3D surface image were applied to other 3D surface images for one patient. We performed the rigid image registration using the polylines defined. Figure 3 shows a sample of 3D surface image registration. The color map indicates the difference between the registered 3D surface images. In each patient group parotid and VOIs on the same side as the location of the tumor were evaluated. The percent weight loss was also evaluated during the treatment course. All changes were evaluated according to the differences in values obtained from weekly images and that obtained from the images of the first week. Spearman’s correlation analysis was performed to investigate the relationship among the above calculated variables.^22^ *P* value was also calculated for each value of the correlation coefficients (*r*) to examine the statistical significance of the values. Correlations between averaged volume changes obtaining from 3D surface images and MRI images were analyzed against the volumetric parameters (PTV, Parotid, VOIs, and Weight) to evaluate the feasibility of the 3D scanner to applicate on clinical practice. In addition, the volume change of PTV and VOIs were evaluated for each patient to identify the appropriate case for clinical application, and the correlation was also calculated during the treatment period.

## 3 RESULTS

### 3.A Changes in structure volume

**FIG. 4:**
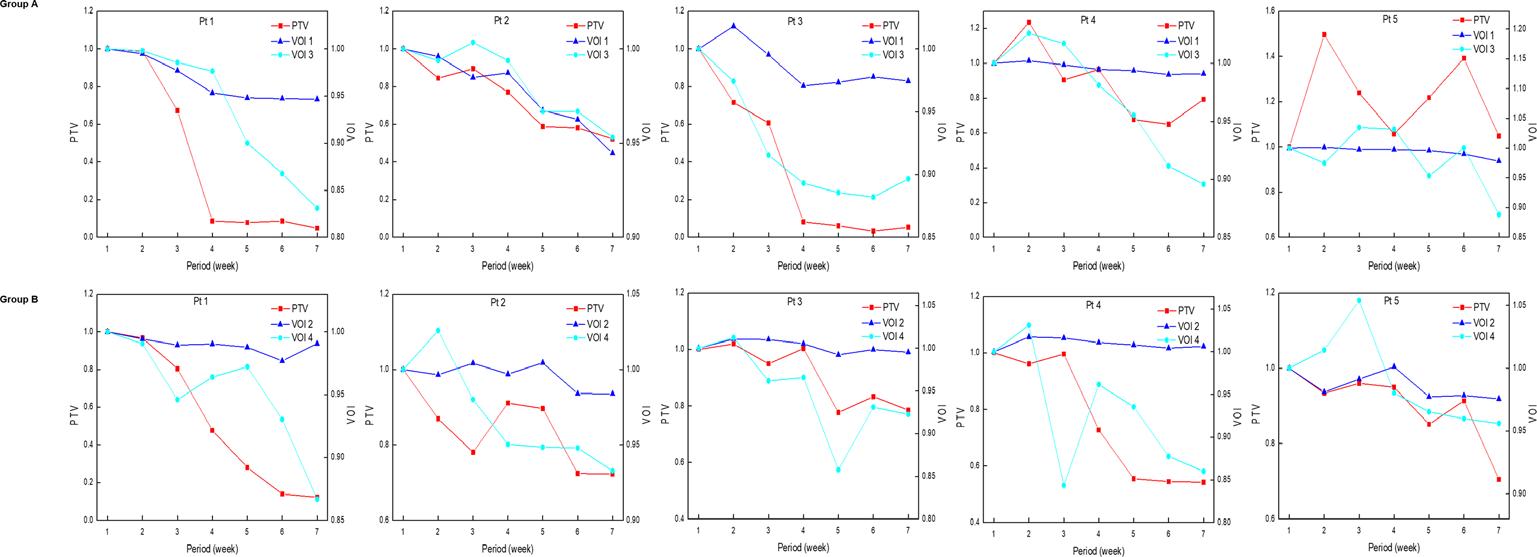
PTV and VOIs volume changes were observed for each patient (pt #) during the treatment course.

**FIG. 5:**
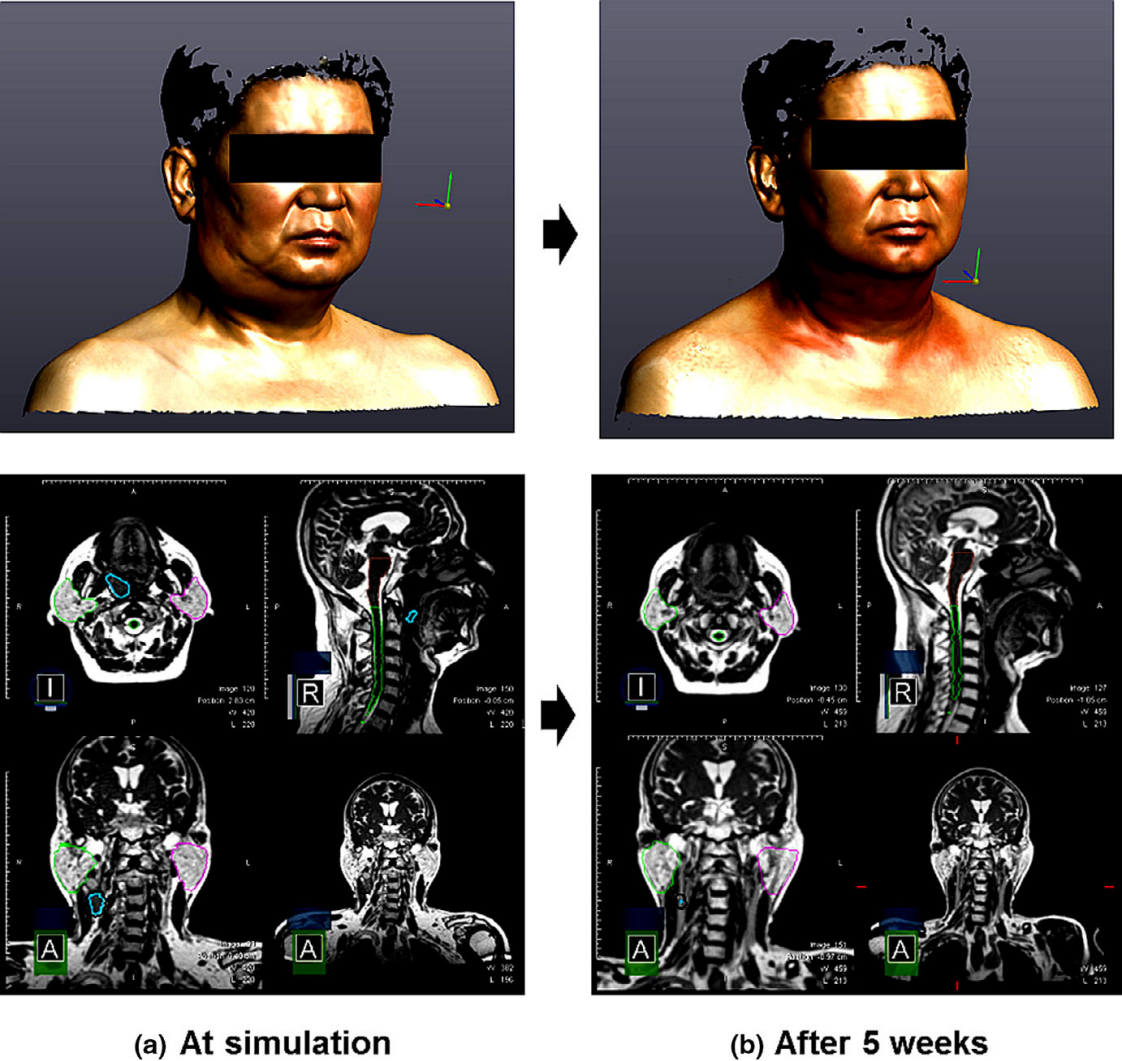
Representative case with right tongue cancer for (a) three‐dimensional (3D) surface imaging and MRI with contour at simulation (b) 3D surface imaging and MRI with contour after 5 weeks. The contours indicate brainstem (brown), spinal cord (green), target (blue), PTV (black), parotid_Rt (lime), parotid_Lt (violet); MRI, magnetic resonance imaging; 3D, three dimensional.

Table 2 summarizes the mean value with standard deviation of the relative change calculated from each patient during the treatment course compared to the first week. The changes in the PTV and parotid volumes (ipsilateral) were observed from the organ structures in the weekly MRI, and VOIs were obtained from the 3D optical scanner. The average PTVs were 89.7 ± 76.1 cc and 60.2 ± 37.0 cc for patient groups A and B, respectively. The PTV changed significantly during the treatment course for both groups. In Group A (right side), the PTV deviation was lager and showed more significant changes than that in Group B (left side). The changes in the averaged PTV in Group A showed a temporary increase of 5.6% after 1 week treatment. At 3 weeks of treatment, the PTV in Group A decreased by 13.6% compared to the initial volume and then showed a reduction of 50.7% after the final treatment. Three tonsil cancer patients showed the largest reduction in PTV after the last treatment at approximately 90% compared to the initial volume in both groups. In Group A, for the change in parotid volume, there was no significant difference between patients, and the deviation was not large. However, the parotid volume change in Group B showed more significant changes than that in Group A. The changes in the averaged parotid volume showed a temporary decrease of 5.5% after 2 weeks of treatment in group B. Subsequently, the averaged parotid volume reduced by up to 3.8% and 28.0% for groups A and B, respectively. The 3D surface images were used to observe the changes in the averaged VOIs separately. In Group A, VOI 1 on the facial area and VOI 3 on the cervical area decreased gradually during the treatment course. Subsequently, the averaged VOIs reduced by up to 3.3% and 10.7% for VOI 1 and VOI 3, respectively. In Group B, only VOI 4 decreased gradually during the treatment course and reduced by up to 9.2%. VOI 3 and VOI 4 on the cervical area changed more than VOI 1 and VOI 2 on the facial area. Figure 4 shows the normalized volume changes of PTV and VOIs for each patient during the treatment course. In Group A, the changes in VOIs tended to be similar overall for patients who showed gradual change in PTV during treatment period. In four out of five patients, VOI 3 decreased gradually similar to changes in PTV. In Group B, VOI 2 did not show the significant change during treatment period in all patients. VOI 4 was changed during treatment period, but two patients showed the strong correlation with the changed in PTV. In particular, one patient with right tongue cancer, as depicted in Fig. 5, showed a significant change in VOI 3 (right cervical area) and MRI, respectively, after 5 weeks of treatment. The volume change was 10% from the initial volume (225.8 cc). Finally, the average weight loss was tracked during the treatment course. All patients showed gradual weight loss in both groups.

**TABLE 2:** Normalized mean value with standard deviation of the relative change calculated from each patient during the treatment course compared to the first week.

Patient group	Parameters	Week2	Week3	Week4	Week5	Week6	Week7
A (Right side)	PTV	−5.6 ± 31.2%	13.6 ± 24.8%	40.8 ± 47.5%	47.5 ± 47.9%	45.2 ± 54.8%	50.7 ± 44.4%
	Parotid_Rt	−13.7 ± 13.7%	2.5 ± 5.5%	3.1 ± 10.8%	0.9 ± 24.8%	−1.4 ± 46.5%	3.8 ± 33.2%
	VOI 1	−0.2 ± 0.9%	0.9 ± 0.9%	1.9 ± 1.9%	2.4 ± 2.0%	2.6 ± 1.9%	3.3 ± 2.1%
	VOI 3	0.7 ± 2.1%	0.9 ± 4.6%	2.5 ± 5.1%	6.8 ± 3.7%	7.4 ± 5.6%	10.7 ± 4.3%
	Weight	0.1 ± 0.2%	0.9 ± 1.1%	0.3 ± 2.0%	1.4 ± 2.0%	3.2 ± 3.1%	4.9 ± 4.3%
B (Left side)	PTV	5.0 ± 5.4%	10.2 ± 9.8%	18.6 ± 21.5%	32.8 ± 25.5%	36.9 ± 30.7%	42.4 ± 26.8%
	Parotid_Lt	0.8 ± 8.3%	5.5 ± 9.8%	10.2 ± 18.1%	17.5 ± 21.2%	17.0 ± 15.9%	28.0 ± 12.1%
	VOI 2	0.0 ± 1.4%	−0.2 ± 1.2%	−0.1 ± 0.8%	0.6 ± 1.3%	1.2 ± 1.2%	1.0 ± 1.2%
	VOI 4	−1.5 ± 1.6%	4.3 ± 7.6%	3.6 ± 1.1%	6.4 ± 4.6%	7.1 ± 3.2%	9.2 ± 4.2%
	Weight	−0.3 ± 0.8%	−0.7 ± 0.8%	1.1 ± 2.8%	3.1 ± 4.1%	3.1 ± 3.6%	4.4 ± 3.7%

PTV, planning target volume; Rt, right; Lt, left; VOI 1, 3D surface volume of right facial area; VOI 2, 3D surface volume of left facial area; VOI 3, 3D surface volume of right cervical area; VOI 4, 3D surface volume of left cervical area.

### 3.B Correlation analysis

Spearman’s correlation coefficients (*r*) with absolute values of averaged volume were calculated for correlation analysis between the volumetric parameters, as summarized in Table 3. In Group A (right side), the change in PTV correlated strongly with right‐side parotid, VOI 1, and VOI 3, respectively. The *r* value between PTV and parotid was 0.825 with statistical significance (*P* < 0.05). The *r* values between PTV and VOI showed strong correlations with statistical significance (*P* < 0.01). The parotid also showed strong correlations with VOIs (*P* < 0.01). The weight loss was not strongly correlated with either PTV or parotid, whereas it was with VOI 1 (*r* = 0.847 with *P* < 0.05) and VOI 3 (*r* = 0.929 with *P* < 0.01). In Group B (left side), the change in PTV correlated strongly as positive with each volumetric parameter, including weight loss. These correlations were statistically significant. There was a strong correlation (*r* = 0.818 with *P* < 0.05) between parotid and VOI 2. With VOI 4, the parotid showed a stronger correlation (*r* = 0.943 with *P* < 0.01). In this group, the weight loss was correlated with other volumetric parameters. Spearman’s correlation coefficients (*r*) with absolute values for each patient were calculated between the PTV and VOIs, as summarized in Table 4. The change in PTV correlated strongly with VOI 1 and VOI 3 in three patients in Group A. One patient only showed the strong correlation between PTV and VOI 3. As shown in Fig. 4, the 5th patient in Group A has no correlation between PTV and VOIs. The PTV also showed more correlation with VOI 4 on the cervical area than VOI 2 on the facial area in Group B.

**TABLE 3:** Averaged correlation coefficients (*r*) among the evaluated volumetric parameters for patient group.

Patient group	Parameters	Parotid	VOI 1 or 2	VOI 3 or 4	WL
A (Right side)	PTV	0.825 (*P* = 0.022)	0.962 (*P* = 0.001)	0.889 (*P* = 0.007)	0.706 (*P* = 0.076)
	Parotid_Rt	–	0.910 (*P* = 0.004)	0.823 (*P* = 0.023)	0.745 (*P* = 0.054)
	VOI 1	–	–	0.958 (*P* = 0.001)	0.847 (*P* = 0.016)
	VOI 3	–	–	–	0.929 (*P* = 0.002)
B (Left side)	PTV	0.981 (*P* < 0.001)	0.873 (*P* = 0.01)	0.926 (*P* = 0.003)	0.937 (*P* = 0.002)
	Parotid_Lt	–	0.818 (*P* = 0.025)	0.943 (*P* = 0.001)	0.952 (*P* = 0.001)
	VOI 2	–	–	0.755 (*P* = 0.05)	0.895 (*P* = 0.006)
	VOI 4	–	–	–	0.844 (*P* = 0.017)

PTV, planning target volume; Rt, right; Lt, left; VOI 1, 3D surface volume of right facial area; VOI 2, 3D surface volume of left facial area; VOI 3, 3D surface volume of right cervical area; VOI 4, 3D surface volume of left cervical area; WL, weight loss.

**TABLE 4:** Correlation coefficients (*r*) between PTV and VOIs for each patient.

Patient group	Parameters (PTV)	VOI 1	VOI 2	VOI 3	VOI 4
A (Right side)	Pt 1	0.893 (*P* = 0.007)	–	0.893 (*P* = 0.007)	–
	Pt 2	0.893 (*P* = 0.007)	–	0.946 (*P* = 0.001)	–
	Pt 3	0.607 (*P* = 0.148)	–	0.893 (*P* = 0.007)	–
	Pt 4	0.929 (*P* = 0.003)	–	0.786 (*P* = 0.036)	–
	Pt 5	0.18 (*P* = 0.699)	–	0.126 (*P* = 0.788)	–
B (Left side)	Pt 1	–	0.607 (*P* = 0.148)	–	0.857 (*P* = 0.014)
	Pt 2	–	0.595 (*P* = 0.159)	–	0.607 (*P* = 0.148)
	Pt 3	–	0.893 (*P* = 0.007)	–	0.964 (*P* < 0.001)
	Pt 4	–	0.179 (*P* = 0.702)	–	0.393 (*P* = 0.383)
	Pt 5	–	0.893 (*P* = 0.007)	–	0.786 (*P* = 0.036)

PTV, planning target volume; Pt, patient; VOI 1, 3D surface volume of right facial area; VOI 2, 3D surface volume of left facial area; VOI 3, 3D surface volume of right cervical area; VOI 4, 3D surface volume of left cervical area.

## 4 DISCUSSION

We investigated the feasibility of optical 3D scanner surface imaging for predicting the volume changes of head and neck cancer patients during treatment. Ten patients with different types of H&N cancer were involved in this feasibility study. It is well‐recognized that all patients are individuals and respond differently to radiation therapy, even if they have the same type of cancer in the same location.^23^, ^24^ Therefore, to achieve these individual therapeutic benefits, we considered the ART treatment plan approach that reflects each anatomical change. However, the ART process is time‐ and resource‐intensive, requires additional imaging, and does not lead to a clinically relevant benefit for all patients. Ahn *et al*. reported their experiences with 23 H&N patients who had prospectively planned rescans at 11, 22, and 33 fractions; 35% of the patients did not receive a dosimetric benefit with ART, which underscores the need for careful selection.^25^ Thus, it is imperative that patients who are likely to require ART are identified and the ideal time when re‐planning is necessary decided.

With no standardization, little data have been published on identifying factors that can predict the need for ART.^8^, ^26^, ^27^ Some studies suggest prognostic factors of shrinkage by weight loss, patient age, and radiation dose.^28^, ^29^, ^30^ However, these results are inconsistent and unclear. Sanguineti et al. investigated a change in volume as predictive factors using weekly CT scans and reported that parotid gland shrinkage during IMRT was not linear.^31^ Accordingly, in this study, the averaged patient data, for patients with the same‐side tumor, were adopted rather than patient‐specific analysis in order to investigate the feasibility of using optical 3D scanner surface imaging to predict volume changes. Based on the correlations between the MR and 3D surface images, the volume changes of PTV and the parotid could be predicted with optical 3D scanner surface imaging. Patient weight loss may be an indicator of adaptive planning. Lee et al. reported that major weight loss above 6% needs adaptive planning.^32^ Duma et al. reported no correlation between weight loss and volume changes of regions of interest (ROIs), except for a strong positive correlation with shrinkage of the parotid.^33^ In this study, weight loss showed a strong positive correlation with the volume changes of ROIs. After the final week of treatment, however, the weight loss was above 4–5%, and it was not a local reduction in the volume of the H&N region. The optical 3D scanner surface images, which were for local regional volume, also showed a strong positive correlation with the volume change of the ROIs. Compared with the first week treatment, the volume changes of VOIs on the facial area showed the decrease of 1%–3% at the final week of treatment. For the VOIs on the cervical area, the volume changes showed the decrease of 9‐10% in this study. These reductions were not significant, but the volume could be measured from the 3D surface image with sub‐cubic millimeter accuracy.

Based on these results, it shows that 3D surface images are useful to monitor the anatomy change and to apply as surface surrogate to trigger ART. Due to benefits from relatively low cost, ease of use, and advantage of no additional radiation exposure, the feasibility of using optical 3D scanners in clinical practice can be considered. Dong et al., used this optical scanner to investigate the surface image registration for image‐guided neurosurgery.^34^ In future works, the number of patients can be increased for the re‐verification of statistical significance, and this methodology can be applied to other treatment regions with large volume changes on the body surface can be carried out. Owing to individual patient responses, much additional work is needed to increase model prediction confidence, including in dosimetric analysis.

## 5 CONCLUSION

In this study, we verified that the volume changes undergone by H&N cancer patients during treatment can be detected by surface imaging using optical 3D scanners. The optical 3D scanner could be applied to track changes in volume without additional radiation exposure.

## CONFLICT OF INTEREST

The authors declare no conflict of interest.

## References

[1] Lee N , Puri DR , Blanco AI , Chao KS . Intensity‐modulated radiation therapy in head and neck cancers: an update. Head Neck. 2007;29:387–400. 16358297 10.1002/hed.20332

[2] Wortel RC , Incrocci L , Pos FJ , et al. Acute toxicity after image‐guided intensity modulated radiation therapy compared to 3D conformal radiation therapy in prostate cancer patients. Int J Radiat Oncol Biol Phys. 2015;91:737–744. 25752386 10.1016/j.ijrobp.2014.12.017

[3] Wu Q , Chi Y , Chen PY , Krauss DJ , Yan D , Martinez A . Adaptive replanning strategies accounting for shrinkage in head and neck IMRT. Int J Radiat Oncol Biol Phys. 2009;75:924–932. 19801104 10.1016/j.ijrobp.2009.04.047

[4] Yan D , Yan S , Wang Q , Liao X , Lu Z , Wang Y . Predictors for replanning in loco‐regionally advanced nasopharyngeal carcinoma patients undergoing intensity‐modulated radiation therapy: a prospective observational study. BMC Cancer. 2013;13:548. 24237861 10.1186/1471-2407-13-548 PMC3840644

[5] Bando R , Ikushima H , Kawanaka T , et al. Changes of tumor and normal structures of the neck during radiation therapy for head and neck cancer requires adaptive strategy. J Med Invest. 2013;60:46–51. 23614911 10.2152/jmi.60.46

[6] Wang Z‐H , Yan C , Zhang Z‐Y , et al.. Radiation‐induced volume changes in parotid and submandibular glands in patients with head and neck cancer receiving postoperative radiotherapy: a longitudinal study. Laryngoscope. 2009;119:1966–1974. 19688858 10.1002/lary.20601

[7] Bhide SA , Davies M , Burke K , et al. Weekly volume and dosimetric changes during chemoradiotherapy with intensity‐modulated radiation therapy for head and neck cancer: a prospective observational study. Int J Radiat Oncol Biol Phys. 2010;76:1360–1368. 20338474 10.1016/j.ijrobp.2009.04.005

[8] Chen AM , Daly ME , Cui J , Mathai M , Benedict S , Purdy JA . Clinical outcomes among patients with head and neck cancer treated by intensity‐modulated radiotherapy with and without adaptive replanning. Head Neck. 2014;36:1541–1546. 23996502 10.1002/hed.23477

[9] Kataria T , Gupta D , Goyal S , et al. Clinical outcomes of adaptive radiotherapy in head and neck cancers. Br J Radiol. 2016;89:20160085. 26986461 10.1259/bjr.20160085 PMC5258178

[10] Surucu M , Shah KK , Roeske JC , Choi M , Small W Jr , Emami B . Adaptive radiotherapy for head and neck cancer. Technol Cancer Res Treat. 2017;16:218–223. 27502958 10.1177/1533034616662165 PMC5616033

[11] Schwartz DL , Garden AS , Thomas J , et al. Adaptive radiotherapy for head‐and‐neck cancer: initial clinical outcomes from a prospective trial. Int J Radiat Oncol Biol Phys. 2012;83:986–993. 22138459 10.1016/j.ijrobp.2011.08.017 PMC4271827

[12] Schwartz DL , Garden AS , Shah SJ , et al. Adaptive radiotherapy for head and neck cancer–dosimetric results from a prospective clinical trial. Radiother Oncol. 2013;106:80–84. 23369744 10.1016/j.radonc.2012.10.010

[13] Srinivasan K , Mohammadi M , Shepherd J . Applications of linac‐mounted kilovoltage cone‐beam computed tomography in modern radiation therapy: a review. Pol J Radiol. 2014;79:181–193. 25006356 10.12659/PJR.890745 PMC4085117

[14] McPartlin AJ , Li XA , Kershaw LE , et al. MRI‐guided prostate adaptive radiotherapy ‐ a systematic review. Radiother Oncol. 2016;119:371–380. 27162159 10.1016/j.radonc.2016.04.014

[15] Acharya S , Fischer‐Valuck BW , Kashani R , et al. Online magnetic resonance image guided adaptive radiation therapy: first clinical applications. Int J Radiat Oncol Biol Phys. 2016;94:394–403. 26678659 10.1016/j.ijrobp.2015.10.015

[16] Gaisberger C , Steininger P , Mitterlechner B , et al. Three‐dimensional surface scanning for accurate patient positioning and monitoring during breast cancer radiotherapy. Strahlenther Onkol. 2013;189:887–893. 23740155 10.1007/s00066-013-0358-6

[17] Wikstrom K , Nilsson K , Isacsson U , Ahnesjo A . A comparison of patient position displacements from body surface laser scanning and cone beam CT bone registrations for radiotherapy of pelvic targets. Acta Oncol. 2014;53:268–277. 23786175 10.3109/0284186X.2013.802836

[18] Walter F , Freislederer P , Belka C , Heinz C , Sohn M , Roeder F . Evaluation of daily patient positioning for radiotherapy with a commercial 3D surface‐imaging system (Catalyst). Radiat Oncol. 2016;11:154. 27881158 10.1186/s13014-016-0728-1 PMC5122202

[19] Krell G , Saeid Nezhad N , Walke M , Al‐Hamadi A , Gademann G . Assessment of iterative closest point registration accuracy for different phantom surfaces captured by an optical 3D sensor in radiotherapy. Comput Math Methods Med. 2017;2017:2938504. 28163773 10.1155/2017/2938504 PMC5253513

[20] Lee NY , Zhang Q , Pfister DG , et al. Addition of bevacizumab to standard chemoradiation for locoregionally advanced nasopharyngeal carcinoma (RTOG 0615): a phase 2 multi‐institutional trial. Lancet Oncol. 2012;13:172–180. 22178121 10.1016/S1470-2045(11)70303-5 PMC4985181

[21] International Organization for Standardization . ISO 10360–2: Geometrical product specifications (GPS) – Acceptance and reverification tests for coordinate measuring machines (CMM) – Part2: CMMs used for measuring linear dimensions; 2009.

[22] Spearman C . The proof and measurement of association between two things. Int J Epidemiol. 2010;39:1137–1150. 21051364 10.1093/ije/dyq191

[23] Yaromina A , Krause M , Baumann M . Individualization of cancer treatment from radiotherapy perspective. Mol Oncol. 2012;6:211–221. 22381063 10.1016/j.molonc.2012.01.007 PMC5528361

[24] Wang W , Lang J . Strategies to optimize radiotherapy based on biological responses of tumor and normal tissue. Exp Ther Med. 2012;4:175–180. 22970024 10.3892/etm.2012.593 PMC3439020

[25] Ahn PH , Chen C‐C , Ahn AI , et al. Adaptive planning in intensity‐modulated radiation therapy for head and neck cancers: single‐institution experience and clinical implications. Int J Radiat Oncol Biol Phys. 2011;80:677–685. 20619553 10.1016/j.ijrobp.2010.03.014

[26] Brown E , Owen R , Harden F , et al. Predicting the need for adaptive radiotherapy in head and neck cancer. Radiother Oncol. 2015;116:57–63. 26142268 10.1016/j.radonc.2015.06.025

[27] Surucu M , Shah KK , Mescioglu I , et al. Decision trees predicting tumor shrinkage for head and neck cancer: implications for adaptive radiotherapy. Technol Cancer Res Treat. 2016;15:139–145. 25731804 10.1177/1533034615572638

[28] Barker JL , Garden AS , Ang KKian , et al. Quantification of volumetric and geometric changes occurring during fractionated radiotherapy for head‐and‐neck cancer using an integrated CT/linear accelerator system. Int J Radiat Oncol Biol Phys. 2004;59:960–970. 15234029 10.1016/j.ijrobp.2003.12.024

[29] Broggi S , Fiorino C , Dell’Oca I , et al. A two‐variable linear model of parotid shrinkage during IMRT for head and neck cancer. Radiother Oncol. 2010;94:206–212. 20117852 10.1016/j.radonc.2009.12.014

[30] Vasquez Osorio EM , Hoogeman MS , Al‐Mamgani A , Teguh DN , Levendag PC , Heijmen BJ . Local anatomic changes in parotid and submandibular glands during radiotherapy for oropharynx cancer and correlation with dose, studied in detail with nonrigid registration. Int J Radiat Oncol Biol Phys. 2008;70:875–882. 18262099 10.1016/j.ijrobp.2007.10.063

[31] Sanguineti G , Ricchetti F , Thomas O , Wu B , McNutt T . Pattern and predictors of volumetric change of parotid glands during intensity modulated radiotherapy. Br J Radiol. 2013;86:20130363. 24029628 10.1259/bjr.20130363 PMC3830435

[32] Lee C , Langen KM , Lu W , et al. Evaluation of geometric changes of parotid glands during head and neck cancer radiotherapy using daily MVCT and automatic deformable registration. Radiother Oncol. 2008;89:81–88. 18707786 10.1016/j.radonc.2008.07.006

[33] Duma MN , Kampfer S , Schuster T , Winkler C , Geinitz H . Adaptive radiotherapy for soft tissue changes during helical tomotherapy for head and neck cancer. Strahlenther Onkol. 2012;188:243–247. 22294198 10.1007/s00066-011-0041-8

[34] Dong Y , Zhang C , Ji D , Wang M , Son Z . Regional‐surface‐based registration for image‐guided neurosurgery: effects of scan modes on registration accuracy. Int J Comput Assit Radiol Surg. 2019;14:1303–1315. 10.1007/s11548-019-01990-6 31055765

